# Editorial

**Published:** 1873-11

**Authors:** 


### Editorial.

### A HISTORY OF AMERICAN DENTISTRY AND DENTAL SURGERY.

A work with the above title is being prepared by the Atlantic Publishing Co., of New York, with the co-operation and assistance of several well-known gentlemen of the profession. It is proposed to embrace careful resume's of the early history of the profession, and its progress to the present time, together with biographical sketches of the representative American dentists.

The desirability and value of such a work, no one will question. The only question that has been asked is, "Will it be a true and faithful work?" In answer to this and similar questions, we will reply that those who have an opportunity to know, regard the publishers as perfectly reliable and disposed to do the best thing for the profession. Similar works prepared by them give promise that this will answer the highest expectations. In addition to this, there is an advisory committee of prominent dentists who are interested in having the work issued in the most correct manner. We doubt not the work will be in every respect acceptable to the profession.

No systematic work of the kind has hitherto been published or prepared, and the call for it is very urgent; time is passing on, and much that is valuable will soon have passed away beyond reach, unless some effort is made for its preservation. Biographies are, perhaps, of less importance than other records. The history of the growth, development and progress of the profession, and the instrumentalities by which these have been obtained, should certainly be written, and put in some tangible and permanent form, that those who come after may know the way by which the present position has been obtained.

We bespeak for this enterprise the hearty co-operation and support of the entire profession of this country.

Perhaps but few biographies will be embraced in this volume; it will be largely occupied by historical matter: but there will be, if proper encouragement is given, future volumes issued. The profession can rely upon this work being one of real merit and value.

### MISSISSIPPI VALLEY DENTAL SOCIETY.

The publication of the proceedings of the last annual meeting of this society, was delayed till the issue of the Oct. No. of the REGISTER. The copy was furnished in proper time, but through changing printers it was lost, and another copy had to be made.

It will be seen by reference to these minutes, that this society has lost none of its vigor through age, but that it seems constantly to be developing and growing. The following was offered and passed unanimously: "*Resolved*, That the members of this Society most earnestly, but respectfully remonstrate against the improper use of the prefix 'Doctor' by dentists, and pledge themselves not to use any title not lawfully belonging to them." This is in the right direction. Every society ought to encourage and foster that which is right, and discourage the wrong in professional matters; so improper has it been deemed for any one to assume the title of Doctor or D. D. S., who has not had the degree conferred upon him by competent authority, that laws in some states have been passed, making it a misdemeanor to do so. Such laws, as well as the above resolution, are based upon the supposition that such titles mean something; that the possessors have accomplished something that others have not; and such title is given as indicating that fact: and if any one may assume it at pleasure, then it ceases to be distinctive or to indicate anything more than mere assumption.

The responsibility of this matter is with the profession, for its members are accustomed to address each other as Doctor, without reference to right. Let such a course be pursued in this matter by the profession, as will place every one in his true position, and not give to one that which does not belong to him, nor degrade, nor detract from another that which is his distinctive right, and is a thing of value to him.

The next meeting of this Society will be about the first of March, in the city of St Louis, Mo., when we hope there will be a large assemblage of the profession.

### MAD RIVER DENTAL SOCIETY.

The semi-annual meeting of this Society was held in Dayton, on the first Tuesday of October. The attendance was good, and there were very interesting sessions held. There were several new topics under consideration that elicited free discussion, and many new thoughts were brought out.

The second subject, viz : "What are the most urgeut demands of the dental profession in its present position ?" Under this head was discussed the importance of office pupilage for students. Its great importance was at once conceded by all, and it was about as readily conceded that there is great failure in this respect by nearly all in the profession who receive students into their offices. The student is permitted to enter the office and learn what he may by limited observation, and by a rambling course of reading, and oftentimes through works not at all adapted to his best interests, the preceptor neither directing nor taking oversight of the study. Usually the opportunity of a student for learning by observation is very limited, except in the laboratory, and even there it is too often confined to making sets of teeth on rubber. The preceptor is very largely responsible for the views the student may entertain in reference to the profession in all particulars. The professional status of the preceptor will usually be perceptible in the student. We hope soon to have the proceedings for publication, and will not here further anticipate their contents. The Mad River is one of the most active of our local societies ; doing its work quietly, but effectively.

### THE EIGHTH ANNUAL MEETING OF THE OHIO STATE DENTAL SOCIETY

Will be held in the Council Chamber in Columbus, commencing on Wednesday, December 3, 1873, at 10 o'clock A. M.

OFFICERS.

*President*, L. Buffett, Cleveland;
*Vice-President*, W. M. Herriot;
*Recording Sec'y*, C. R. Taft, Mansfield;
*Cor. Sec'y*, C. R. Butler, Cleveland;
*Treasurer*, I. Williams, New Philadelphia;
*Executive Committee*, H. A. Smith, A. F. Emminger, D. R. Jennings.

ORDER OF BUSINESS.

1. Calling of the roll.
2. Reading of the minutes.
3. Reports of standing committees.
4. Reports of special committees.
5. Election of members and payment of dues.

SUBJECTS FOR ESSAYS AND DISCUSSIONS.

1. What are the benefits to be derived from a proper care of the deciduous teeth? Description of methods of treatment.
2. How much is the success of filling teeth due to the mere manipulation?
3. Observations on the methods of operating, and materials used in filling teeth.
4. Why has not dentistry received the general recognition as a specialty of medicine that it is justly entitled to?
5. Dental legislation: its present status in the State of Ohio. Is a modification in any respect desirable?
6. Conservative treatment of exposed or diseased pulps. What are the best materials for filling roots of teeth?
7. Irregularities in position of the permanent teeth: their cause and mode of treatment.
8. Artificial dentures. What are the principles to be observed in their construction?
9. Does the administration of the lime phosphates aid in the development of good teeth?

The Executive Committee would respectfully urge the attendance of members upon this session of our State Society. A cordial invitation is also extended to dentists not members, to attend this meeting.

### DIED

Sept. 23, 1873, at her residence, Belmont Place, near Newport, Ky., Mrs. Belle P. Taylor, wife of Dr. James Taylor, in the 45th year of her age.

The deceased was married Feb. 29, 1860, to Dr. James Taylor, who has been a prominent member of the dental profession for many years.

Mrs. Taylor was a woman of rare accomplishments. Her mind was cultivated. Her literary tastes were refined. She read the works of the best authors, and possessed varied stores of knowledge. These, with her genial disposition and cultivated manners, made her the charm of the social circle and the light and joy of her home.

Those who knew her best saw her Christian character ripen and her trust increase from year to year, and during the last year or two she seems to have searched her heart and fixed her faith on Christ more firmly than ever.

In her last sickness her faith shone out triumphantly. She was calm and serene. Her mind was full of peace. Her heart was stayed on God. She wished only to do and suffer the will of God, whether by life or by death.

> The star that sets
> Beyond the western wave is not extinct;
> It brightens in another hemisphere,
> And gilds another evening with its rays.

---

At his residence, in Chillicothe, Ohio, on the morning of October 29, at 20 minutes past 12 o'clock, Hervey, eldest son of Dr. and Mrs. H. Scott, aged 30 years and 6 months.

